# Access and Participation of Students with Disabilities: The Challenge for Higher Education

**DOI:** 10.3390/ijerph191911918

**Published:** 2022-09-21

**Authors:** José María Fernández-Batanero, Marta Montenegro-Rueda, José Fernández-Cerero

**Affiliations:** Department of Teaching and Educational Organization, University of Seville, 41013 Seville, Spain

**Keywords:** barriers, facilitators, higher education, inclusion, disability, inclusive research

## Abstract

Access to university is a right for all people; however, access to higher education for people with disabilities is still a challenge. The present study, based on a systematic review of the literature, aims to report on the challenges faced by students with disabilities in accessing and participating in higher education. The systematic review of four databases resulted in 20 studies published between 2011 and 2021. The results indicate that students with disabilities face numerous challenges in accessing university education. Based on the results, strategies are proposed in order to provide equal opportunities and success in higher education for students with disabilities.

## 1. Introduction

The UNESCO conference in Salamanca (1994) had an impact not only on educational thought, policy, and practice, but also on culture [1]. Today, it continues to present an indispensable point of reference for all those involved in the struggle for inclusive education. This legacy immersed in the digital age is leading educational institutions and professionals to a profound transformation and a radical change in their ways of doing, acting, and training. In the framework of the European Higher Education Area, a more inclusive character is being demanded from the University, as evidenced in different international declarations [2]. Furthermore, Sustainable Development Goal (SDG) 4 on education of the European Agenda 2030 calls for ensuring an inclusive and equitable quality education and promoting lifelong learning opportunities for all by 2030. It emphasises the importance of inclusion and equity as the foundation for quality education and learning.

In the case of persons with disabilities, the European Agency for Special Needs, and Inclusive Education [2] and the United Nations High Commissioner recognised inclusive education as an opportunity for their empowerment [3], as well as an opportunity to remove barriers to learning and participation for all learners [4]. However, at present, practices of educational exclusion and discrimination are still present in all education systems, constituting real barriers or obstacles to progress [5].

## 2. Conceptualisation

The scientific literature shows that there is a wide range of definitions around access and participation of students with disabilities in higher education. Thus, according to the World Health Organisation [6], barriers to inclusion are all those physical, social, and attitudinal factors that prevent or limit the full realisation of individuals. Authors such as Ainscow [1] refer to them as obstacles to inclusion that hinder or limit learning, belonging and participation, under equal conditions, in educational processes. Authors such as Darrow [7] classify barriers in three areas: organisational, attitudinal, and knowledge barriers. The first ones refer to the way in which institutions and classes are structured, how the objectives proposed to students with disabilities are defined, how teaching strategies are used and how classes are managed.

Attitudinal barriers relate to the beliefs and attitudes that teachers may have about educational services for students with disabilities, including curricular adjustments, interactions with students, and participation in the institution and community activities.

Conversely, aids, supports, or facilitators are elements of the educational context that contribute to students’ social and educational inclusion in educational contexts [8]. Within the studies referring to the school environment, Pivik, McComas and Laflamme [9] identify three aspects to be addressed as facilitators: environmental modifications, changes in policies, and institutional resources. Regarding environmental modifications, they consider it important to include technological resources and to adapt the infrastructure to the needs of the students, and about policies, they recommend educating the population and making curricular adaptations.

On the other hand, participation is a multidimensional concept made up of three interdependent subdimensions. Firstly, it refers to feeling a sense of belonging or the perception of emotional well-being resulting from an established social and academic self-esteem. It also symbolises being part of a peer group, where students are valued and recognised and where identities are constructed in a positive way and not deficient or of lesser value than any other student. Finally, it means taking part in the formal and informal bodies and structures of educational participation [10].

In short, barriers and facilitators constitute one of the different ways of approaching the inclusion (and exclusion) of people with disabilities in higher education. Their effects are the result of the convergence between collective actions, individual actions, and social conditions, and are manifested in different dimensions of students’ academic and social life [11].

## 3. Results, State of Play, Access, and Participation of Students with Disabilities in Higher Education

Many efforts have been made to try to create an educational culture where students feel competent, valued, and not excluded, regardless of their characteristics, interests, abilities, or difficulties. In this sense, access to university for people with disabilities is a legally recognised right [12]. Despite this, there are still legal gaps in its implementation, contributing to the fact that the path of these institutions towards inclusion is increasingly long [13]. We are aware that there is gradually a greater commitment on the part of universities to move towards this objective; despite this, works and studies that give students a voice conclude that universities become an obstacle course that, on many occasions, generates a premature abandonment of university studies [14,15]. On the contrary, it should be noted that students with disabilities recognise the value of universities for their social and educational inclusion, but at the same time they consider that their experiences in this institution are not always positive [15]. Therefore, it is not enough to guarantee access, but rather it is necessary to establish policies and plans to ensure that all students, including those with disabilities, remain and succeed in university studies [16].

Along these lines, in recent years, studies have focused on the different barriers encountered by students with disabilities during their time at university. However, the most common barriers include architectural barriers, lack of information, inaccessible technologies, or regulations that are not applied, as well as teachers. Regarding the latter, teachers are identified as the main obstacle to inclusion [17], as their attitude towards people with disabilities is essential to facilitate student learning [18,19]. Other research focuses on the teacher profile, especially on personal competences as essential values for working in inclusive contexts [20,21]. Studies that have given a voice to inclusive teachers have concluded that when it comes to facilitating the learning of students with disabilities, the diversity of active and participatory methodological strategies where students are included, more affective and emotional, is just as important [22].

Another line of research in relation to the possible barriers encountered by students with disabilities focuses on the teaching and learning processes themselves [23]. These studies show how reasonable adjustments to the curriculum (flexible timing and methodological strategies) to help students participate in the teaching and learning processes on an equal footing with their peers can contribute to the retention and success of students with disabilities [23,24]. Another key element of educational projects that concerns both students and teachers is the assessment tests. Research addressing this issue points to the difficulty for teachers to adjust, especially in examinations. Studies coincide in pointing out the lack of receptiveness of teachers to enable different modes of assessment [25].

Another relevant finding is the importance of peer relationships. Peer support would favour the participation of students with disabilities, as they value the support of their peers as a facilitator of their inclusion in the academic context [24].

This context of access, barriers, and participation of students with disabilities in higher education is where our work is directed, hence the purpose of this article was to analyse the latest research on access and participation of students with disabilities in higher education and the main themes that guide the different studies conducted in this area to deepen the understanding of the challenges of access to university. Therefore, the current analysis aimed to develop a systematic review to answer the following research questions:

Q1. What is the current state of research in the field of students with disabilities in higher education?

Q2. What are the barriers to access and participation of students with disabilities in higher education?

Q3. What aspects could be addressed to facilitate the inclusion of students with disabilities in university education?

## 4. Method

This study adopted the methodology of a systematic literature review [26]. This method allows synthesising the relevant information available on the selected topic [27]. To this end, this study relied on the PRISMA (Preferred Reporting Items for Systematic Reviews and Meta-Analyses) Statement [28] to guide the search, selection, and analysis of data.

### 4.1. Search Strategy

The current study was conducted in July 2022. Four databases (Web of Science (WoS), Scopus, Education Resources Information Center (ERIC), and Google Scholar) were searched, selecting papers published in the last ten years.

Keywords related to students with disabilities in higher education were used as search terms in the title, abstract, and/or keyword fields. The search strategy, according to the particularity of each database, was as follows: (“student with disabilities”) AND (“higher education” OR “university”) AND (“access” OR “participation” OR “inclusion” OR “experience” OR “admission”).

### 4.2. Selection Criteria

The inclusion criteria established for the selection of articles were as follows: (a) empirical articles published in peer-reviewed journals; (b) published in the last ten years, between 2011 and 2021; (c) the sample was students with disabilities in higher education; (d) contained details on access and participation of students with disabilities in higher education. The exclusion criteria were: (a) type of document: reviews, essays, books, book chapters, or conference proceedings, (b) duplicate documents.

### 4.3. Literature Selection

**Figure 1:**
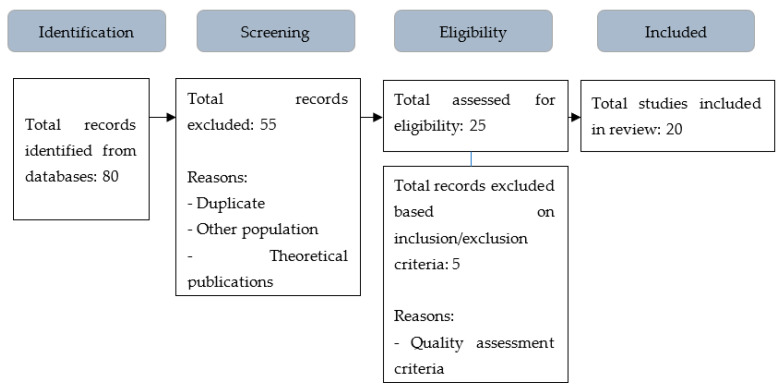
Flow chart of the selection process.

The initial search revealed 80 studies in the different databases analysed. Once the publications had been selected, a thorough review of the titles and abstracts of the selected studies were carried out to exclude those that were duplicated, did not target students with disabilities, or were theoretical studies, eliminating a total of 55 studies. The remaining studies (*n* = 25) were read in full by two authors who checked that they met the inclusion criteria set for this systematic review. Discrepancies between authors were resolved with the third author, thus excluding 5 studies. Therefore, 20 studies were identified for this systematic review. Figure 1 shows the literature search process.

### 4.4. Data Extraction and Analysis

For the extraction and analysis of the data from the articles, a table was developed to extract information from each of the studies: (a) author, (b) year of publication, (c) method, (d) type of disability, (e) country, and (f) main topic or category. Network mapping analysis techniques were also employed using VOSviewer software version 1.6.16. (Centre for Science and Technology Studies, Leiden University, Leiden, The Netherlands) [29] to analyse the themes of the studies included in the review.

## 5. Results

**Figure 2:**
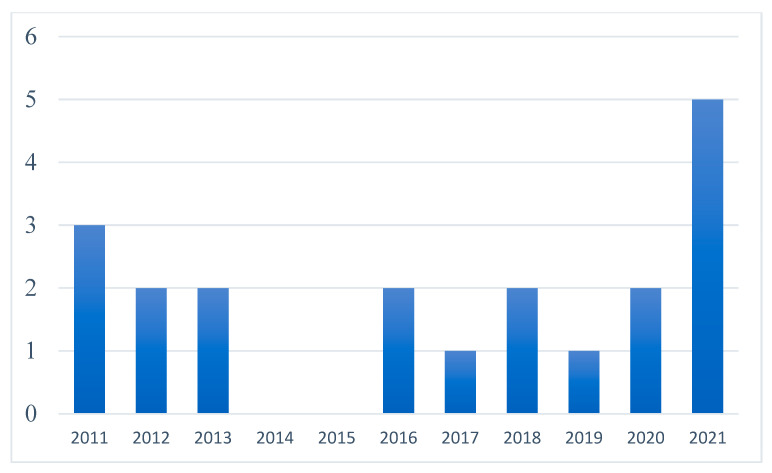
Studies by year of publication.

Considering the search strategy and selection criteria, 20 empirical articles published between 2011 and 2021 were selected for this systematic review indexed in WoS, Scopus, ERIC, and Google Scholar. Considering the year of publication (Figure 2), even though research in this field has been constant over the last ten years, most of the published studies were found in the last year as can be seen in Table 1. Likewise, if we look at the method employed, fourteen of the publications analysed predominantly used a qualitative methodology (*n* = 14, f = 70%), the remaining six were carried out using a quantitative approach (*n* = 6, f = 30%).

**Figure 3:**
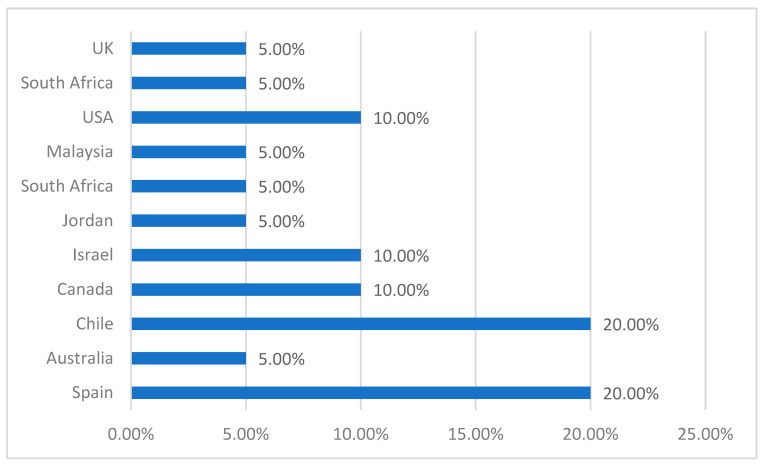
Studies by country of publication.

If we focus on the country of publication, we can see that most of the papers have been carried out in Chile (*n* = 4, f = 20%) and Spain (*n* = 4, f = 20%). Other countries with the highest production included Canada (*n* = 2, f = 10%), South Africa (*n* = 2, f = 10%), Israel (*n* = 2, f = 10%), and the USA (*n* = 2, f = 10%), and to a lesser extent Australia (*n* = 1, f = 5%), Jordan (*n* = 1, f = 5%), Malaysia (*n* = 1, f = 5%), and the UK (*n* = 1, f = 5%) (Figure 3).

**Figure 4:**
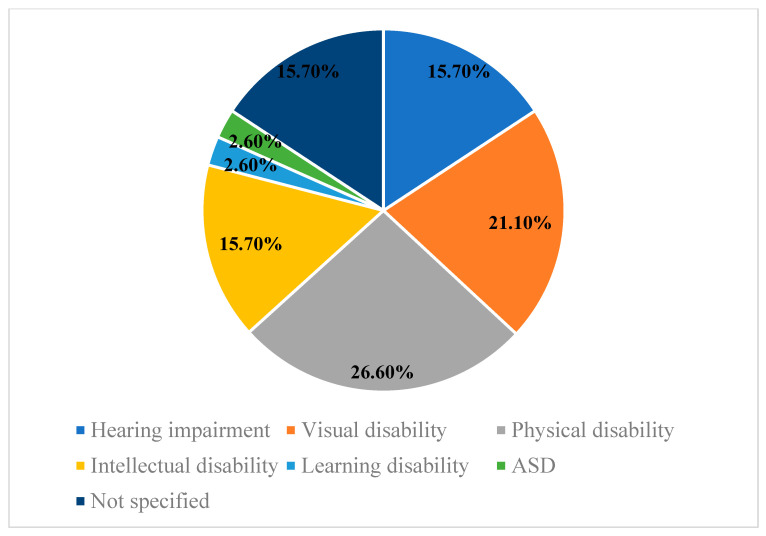
Studies by type of disability.

The selected studies were conducted with students with disabilities in higher education. However, there were differences between the participating sample. Most of the published studies focused on students with physical disabilities (*n* = 10, f = 26.6%). However, the least researched students were students with an autism spectrum disorder (ASD) (*n* = 1, f = 2.6%) and students with learning disabilities (*n* = 1, f = 2.6%). Although most of the research described the type of disability of their participants, there were 15.7% of publications that did not differentiate between the type of disability of their students and investigated the access and participation difficulties of university students with disabilities in general (Figure 4).

Promoting the educational inclusion of students with disabilities in higher education is a challenge. However, in order to make further progress on this path, we need to know what the challenges are that make it impossible for them to access higher education. Thus, this systematic review allowed us to identify the different limitations posed by the research analysed. To this end, we classified the studies according to the main subject of the study. In this sense, we found two categories: eleven studies (61.11%) focused on the obstacles that universities face for the access and participation of these students, and seven studies (38.8%) focused on the factors that facilitate their access to higher education.

**Figure 5:**
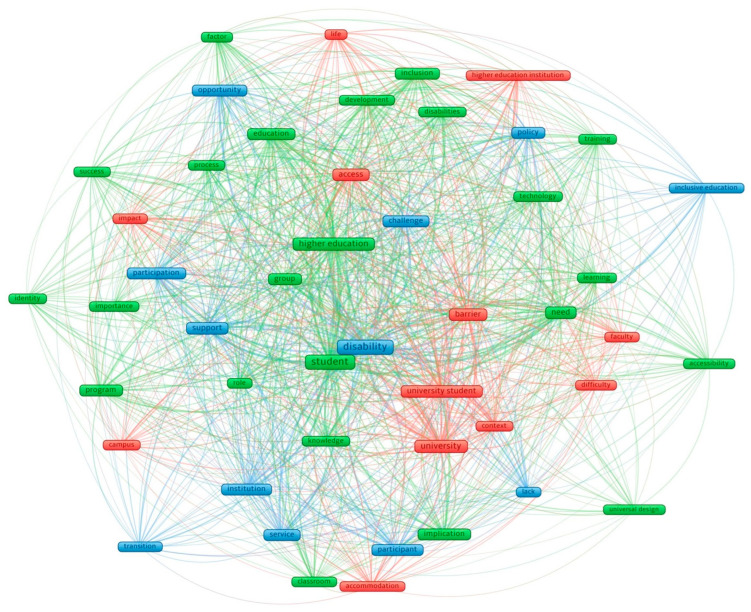
Network of extracted keywords.

In addition, in order to identify the main trends affecting access to higher education for students with disabilities, a keyword analysis was carried out by representing the keywords in a keyword graph (Figure 5). The studies included in the review were loaded, obtaining a total of keywords. After analysing their homogeneity, three thematic clusters were automatically generated according to the degree of similarity of the keywords. Thus, the main challenges or challenges of access and participation of students with disabilities in higher education focused on three clusters:

Cluster 1 (red), related to infrastructure. The infrastructure category was related to the barriers to access to higher education. This cluster grouped items such as barrier, access, context, impact, and campus.

Cluster 2 (green), related to the teaching–learning process. The category was related to barriers involving educational materials, access to information, and teacher training. This cluster grouped items such as students, universal design, process, learning, and training.

Cluster 3 (blue), related to the management of the university institution. The category of institutional management encompassed all those aspects that were not related to the teaching–learning process or at the infrastructure level. It focused on all those measures that favoured the access and inclusion of these students to university education. This cluster grouped together items such as support, institution, transition, service, and policy.

## 6. Discussion

The aim of this systematic review was to analyse the studies published in the last decade on the access and participation of students with disabilities in higher education, responding to the research questions posed.

In response to the first research question, the results showed that there was a high tendency towards qualitative rather than quantitative research in this field, as well as a greater publication of studies carried out in institutions in Chile and Spain, coinciding with previous studies [50]. This may be due to the fact that these countries have participated very actively in the development and implementation of the new international agenda, especially in the definition of Sustainable Development Goal 4, aimed at ensuring inclusive, equitable, and quality education and promoting learning opportunities for all [2,3]. Likewise, even though publications in this field have been increasing over the years, it was evident that it was in the year 2021, where this subject had the greatest boom in the last decade.

About the diversity of students who participated in the works reviewed, it was observed that the samples were mainly of students with physical disabilities. However, there were also numerous studies that focus on students with hearing, visual, and intellectual disabilities. This may be since there is a higher prevalence of students with physical, hearing, visual, and intellectual disabilities in universities [50].

Likewise, after analysing the main topic addressed in each of the studies, two categories were differentiated: those that addressed the obstacles to access and participation of students with disabilities in higher education and, on the other hand, those that addressed the factors that facilitated their success in university inclusion.

In this sense, answering the second research question, we can summarise that the barriers to access and participation of these students were concentrated in three main areas:

- Infrastructure: Students with disabilities present educational needs that must be addressed for them to successfully access education, as the existence of these barriers can impede accessibility for these students [35,45,51]. Architectural or infrastructural barriers are the most common access barriers for students with disabilities. This may be since university facilities are mostly old buildings, therefore, their spaces are not adapted to the needs of students [50], affecting their mobility.
- Teaching–learning process: Studies highlight several barriers to learning. Among them, the lack of preparation of teachers to use a methodology that promotes inclusion in the classroom according to the needs of their students stands out [39]. These results coincide with other studies that have been carried out on the lack of teacher training to cater for these students in higher education [52,53]. They also mention the difficulties of access to material resources, since in most cases they are not adapted to their needs or are limited [34,40,46,54].
- Institutional management: Students highlight that the provision of services to address the queries and needs of students with disabilities are scarce at the university level [44,55], as well as the lack of funding for support programmes for students with disabilities [32].
- In this line, and to answer the third research question posed, the way to facilitate a successful access to university education for these students, the following aspects must be addressed:
- Infrastructure: Students with disabilities demand multiple supports related to access to higher education, mainly related to access and mobility on campus. The elimination of the different architectural barriers, such as the absence of spaces reserved for people with disabilities, the absence of ramps, inadequate signage, or acoustic barriers in classrooms, will facilitate the movement and permanence of these students at the university [30,42,48].
- Teaching–learning process: It is necessary to generate a new organisational response in the attention to diversity and in teacher training [33,49]. Current trends in education point out that all students can be included in education through inclusion programmes, despite their educational needs, offering different opportunities to these students [55], promoting methodological changes in university institutions, and fostering inclusive education. Among these, the incorporation of Universal Design for Learning stands out to increase the participation of these students [50], as most of the resources and materials are not adapted to their needs. This would allow them to work with the rest of their classmates. Recent studies highlight the incorporation of information and communication technologies as potentially beneficial tools for the inclusion and participation of students with disabilities [36].
- On the other hand, to promote the training of teaching staff in the acquisition of competences to cater for the diversity of their students, training courses, and the modification of the specific training plans that are developed in the different universities are necessary [56], which are usually scarce or nonexistent.
- Institutional management: All students present difficulties during the educational process, therefore, it is necessary to provide assistance services for students with disabilities, in order to offer specialised support and guidance to these students [44]. Thus, assistance services for students with disabilities should be created in all university institutions, or at least, the possibility for all students who need it to have a person or scholar to help them with their integration into the university [31,47].

Likewise, although support throughout the educational process is necessary, it is also essential to provide support during the transition from secondary to higher education by establishing transition strategies, as all students require support. Therefore, it is not only necessary to make changes in the academic aspect, but also deep cultural changes that achieve inclusion, developing clear educational policies, establishing economic funds, as well as establishing protocols of good practices to achieve inclusion [37,38,43].

The inclusion of all these aspects has beneficial results for all these students, allowing them to successfully complete their studies, as well as enter the workforce [57].

## 7. Conclusions

This systematic review showed that there were many barriers that limited access to higher education for students with disabilities. Through this systematic review, we learned that, in the last decade, the difficulties of access to higher education for students with disabilities have been studied. However, ten years later, despite the considerable increase in the presence of these students in university classrooms [41] and the strategies developed so far [58], the full inclusion of these students has not been achieved. For this reason, different aspects related to infrastructure, the teaching–learning process and institutional management are necessary to facilitate the presence of these students in university classrooms. Only by improving the accessibility of higher education institutions, training university teaching staff, and raising the awareness of the educational community for inclusive education in higher education will it be possible to promote the success of students with disabilities in university education.

Finally, the implementation of teaching practices based on the principles of Universal Design for Learning could mean in the future the elimination of barriers to learning, not only for students with functional diversity, but also for other students. We are aware that improving teaching practices for students with disabilities will have a positive impact on both teaching and learning for all students.

### Limitations and Future Studies

Among the limitations of our study, it should be noted that the data were extracted from scientific articles published in peer-reviewed journals addressing facilities and barriers to access and participation in higher education for students with disabilities. Future studies would also need to widen the scope to other types of papers. Future research would also need to examine the perceptions of students at other levels of education to understand the differences in their specific needs for a successful access to education.

## Figures and Tables

**Table 1:** Description of the studies included in the review.

Study	Year	Method	Disability Type	Country	Main Topic
Moriña Díez and Molina Romo [30]	2011	Qualitative	Hearing, visual, physical, and intellectual disabilities	Spain	Barriers to university access
Nava-Caballero [31]	2011	Qualitative	Hearing, visual, physical, and intellectual disabilities	Spain	Facilitating factors in access and adaptation
Ryan [32]	2011	Qualitative	Not specified	Australia	Facilitating factors in access and adaptation
Ocampo González [33]	2012	Quantitative	Not specified	Chile	Barriers to university access
Opini [34]	2012	Qualitative	Not specified	Canada	Barriers to university access
McEwan and Downie [35]	2013	Quantitative	Intellectual disability	Canada	Barriers to university access
Zubillaga del Río et al. [36]	2013	Quantitative	Not specified	Spain	Facilitating factors in access and adaptation
Kendall and Tarman [37]	2016	Qualitative	Hearing impaired	UK	Facilitating factors in access and adaptation
Palma et al. [38]	2016	Qualitative	Hearing, visual, physical, and intellectual disability	Chile	Facilitating factors in access and adaptation
Heiman et al. [39]	2017	Quantitative	Not specified	Israel	Facilitating factors in access and adaptation
Alsalem and Abu Doush [40]	2018	Qualitative	Not specified	Jordan	Barriers to university access
Majoko and Dunn [41]	2018	Qualitative	ASD, physical, hearing, and visual disability.	South Africa	Facilitating factors in access and adaptation
Rodríguez Molina & Valenzuela Zambrano [42]	2019	Qualitative	Physical, visual disability, and ASD	Chile	Barriers in access to university
Ansay and Moreira [43]	2020	Qualitative	Physical disability	Chile	Barriers in access to university
Yusof et al. [44]	2020	Qualitative	Physical and visual disability	Malaysia	Barriers to access and adaptation
Braun & Naami [45]	2021	Qualitative	Physical disability	USA	Barriers in access to university
Dreyer [46]	2021	Qualitative	Learning disability	South Africa	Barriers in access to university
Newman et al. [47]	2021	Quantitative	Intellectual disability and hearing impairments	USA	Barriers in access to university
Shpigelman et al. [48]	2021	Qualitative	Physical, visual, hearing, and intellectual disabilities.	Israel	Barriers in access to university
Valle-Flórez et al. [49]	2021	Quantitative	Hearing, visual, physical, and intellectual disabilities.	Spain	Facilitating factors in access and adaptation

## Data Availability

Not applicable.
